# A new plasmid carrying mphA causes prevalence of azithromycin resistance in enterotoxigenic *Escherichia coli* serogroup O6

**DOI:** 10.1186/s12866-020-01927-z

**Published:** 2020-08-11

**Authors:** Ying Xiang, Feng Wu, Yinghui Chai, Xuebin Xu, Lang Yang, Sai Tian, Haoran Zhang, Yinxia Li, Chaojie Yang, Hongbo Liu, Shaofu Qiu, Hongbin Song, Yansong Sun

**Affiliations:** 1 Academy of Military Medical Sciences, NO.20 Dongda Street, Fengtai District, Beijing, 100071 China; 2 Chinese PLA Center for Disease Control and Prevention, NO.20 Dongda Street, Fengtai District, Beijing, 100071 China; 3 Center for Disease Control and Prevention of Southern Theatre Command, Guangzhou, China; 4 Shanghai Municipal Center for Disease Control and Prevention, Shanghai, China

**Keywords:** Enterotoxigenic *Escherichia coli*, Azithromycin, Plasmid, *mphA*, Whole-genome sequencing, Nanopore sequencing

## Abstract

**Background:**

At present, azithromycin has become an effective treatment for severe diarrhea caused by Enterotoxigenic *Escherichia coli* (ETEC) infection. However, enterobacteria have begun to develop resistance to azithromycin and have attracted attention in recent years. This study conducted to described the emergence of a high proportion of azithromycin-resistant ETEC serogroup O6 strains in Shanghai and to analyzed the mechanisms of azithromycin resistance.

**Results:**

Strains from adult diarrhea patients with ETEC serogroup O6 infections were collected by Shanghai Diarrhea Surveillance Network and the Foodborne Surveillance Network from 2016 to 2018. We tested 30 isolates of ETEC O6 serogroup, 26 of which were resistant to azithromycin. Phylogenetic analysis revealed that these ETEC serogroup O6 strains have formed an independent dominant clone. S1-PFGE and southern blotting revealed the presence of the *mphA* gene on the 103 kb plasmid. Illumina and Nanopore sequencing and plasmid coverage analysis further confirmed that azithromycin-resistant strains carried a novel IncFII plasmid harboring *mphA* and *bla*TEM-1 resistance genes.

**Conclusions:**

This is the first study to report a high proportion of azithromycin resistance in a particular ETEC serogroup due to a specific plasmid carrying *mphA*. Our findings indicate the rapid spread of azithromycin resistance, highlighting the urgency of stringent surveillance and control measure.

## Background

Enterotoxigenic *Escherichia coli* (ETEC) is a major cause of infantile and adult diarrhea and traveller’s diarrhea around the world, especially in developing countries [1]. ETEC has two specific virulence factors, colonization factors (CFs) and enterotoxin. CFs, which play important roles in the pathogenesis, are fibrous or roof surface structure that may mediate adhesion to the epithelial cells of the small intestine, being the first and critical step in establishing ETEC infection [2]. ETEC is defined by its ability to generate heat-labile toxin (LT) and/or heat-stable toxin (ST; includes two subtypes STh and STp, STh is human, STp is pig source). ETEC can express a variety of O antigens, of which more than 100 types are associated with clinical ETEC isolates [1, 3]. The O6 serogroup, one of the major serogroups in ETEC, is the dominant serogroup of ETEC in China. Moreover, it has shown an upward trend in recent years [4–6].

Since 1980s, increased antibiotic resistance of ETEC have been reported. In recent years, the most common antibiotic resistance profile of ETEC is resistance to nalidixic acid, ampicillin, sulfonamides and tetracycline, and there have been more and more reports on multidrug resistance of ETEC [5, 7–9]. Because antimicrobial resistance has increased over time and in many areas these antimicrobial agents are losing their usefulness, azithromycin and fluoroquinolones have become first line drugs for the treatment of ETEC infections. Azithromycin, an antimicrobial agent belonging to the macrolide family, owns a favorable membrane permeability, making it very effective in treating enterobacteria infection [10, 11]. Amounts of studies revealed that although several *Enterobacteriaceae* have developed resistance to azithromycin, the overall level of resistance is absolutely low. In the case of *E. coli*, the resistance rate is basically between 0 and 30% [7, 8].

So far, possible molecular mechanisms for the azithromycin resistance have been described, including (1) overexpression of efflux pump, (2) peptidyl tRNA hydrolase overexpression, (3) chromosomal mutations such as changes in ribosomal proteins and 23S rRNA mutations, (4) methylation mediated by methylases encoded by *erm* genes (especially *ermA* and *ermB*), (5) macrolides-inactivation, mediated by esterases encoded by the *ereA* and *ereB* genes and/or phosphotransferases encoded by *mphA* and *mphB* [10–14]. Among these mechanisms, macrolide resistance gene *mphA* was reported to play an important role in developing resistance to azithromycin, and *Enterobacteriaceae* with MIC values more than 256 mg/L of azithromycin often carry *mphA* [10, 15].

To date, research results have shown that ETEC strains without any specific serogroup are highly resistant to azithromycin. However, in a molecular study of ETEC in an adult diarrhea case surveillance network (2016–2018), we found that ETEC serogroup O6 strains were highly resistant to azithromycin, 26 of 30 strains were resistant to azithromycin. Therefore, the purpose of this study was to analyze the antibiotic resistance of 30 strains of ETEC serogroup O6 and study the mechanism of azithromycin resistance.

## Results

### Identification of ETEC strains

A total of 84 adult cases (each ETEC strain corresponded to a different case) eligible for sampling reported by the Shanghai Diarrhea and Foodborne Surveillance Network from 2016 to 2018 were derived from 6 sentinel hospitals in separate districts of Shanghai: Renji Hospital of Pudong New District, People’s Hospital of Putuo District, Sixth People’s Hospital of Xuhui District, Zhongshan Hospital Qingpu Branch of Qingpu District, First People’s Hospital Baoshan Branch of Baoshan District, Ping’an Health Hospital of Fengxian District. The results of serogroup identification showed that among the 84 ETEC strains, except for 3 that could not be grouped, there were 30 strains of O6 serogroup, 22 strains of O25 serogroup, 20 strains of O159 serogroup, 6 strains of O153 serogroup, 2 strains of O15 serogroup and 1 strain of O160 serogroup. All 30 ETEC O6 strains were identified as the research objects, including 5 strains from 2016, 9 strains from 2017, and 16 strains from 2018 (Additional file 1 Table S1). These 30 strains can generate STh, and two of them can also generate LT (Additional file 3 Figure S1).

### Antimicrobial susceptibility testing

Among the 30 strains, 1 (3.3%) were resistant to ceftriaxone and ceftiofur, 2 (6.7%) were resistant to tetracycline, streptomycin and trimethoprim/sulfamethoxazole, 26 (86.7%) were resistant to ampicillin and azithromycin, and 29 (96.7%) were resistant to nalidixic acid. According to the antibiotic resistance profile, 26 strains (86.7%) were classified as multidrug-resistant (MDR) [16]. And all isolates are sensitive to the following 6 antibiotics: cefoxitin, gentamicin, chloramphenicol, ciprofloxacin, sulfisoxazole and amoxicillin/clavulanic acid (Table 1).

**Table 1:** Antimicrobial resistance of 30 ETEC serogroup O6 strains

Antibiotic	R [N(%)]	I [N(%)]	S [N(%)]
Ceftriaxone	1 (3.3)	0 (0.0)	29 (96.7)
Tetracycline	2 (6.7)	0 (0.0)	28 (93.3)
Ceftiofur	1 (3.3)	0 (0.0)	29 (96.7)
Cefoxitin	0 (0.0)	0 (0.0)	30 (100.0)
Gentamicin	0 (0.0)	0 (0.0)	30 (100.0)
Ampicillin	26 (86.7)	0 (0.0)	4 (13.3)
Chloramphenicol	0 (0.0)	0 (0.0)	30 (100.0)
Ciprofloxacin	0 (0.0)	1 (3.3)	29 (96.7)
Trimethoprim/sulfamethoxazole	2 (6.7)	0 (0.0)	28 (93.3)
Sulfisoxazole	0 (0.0)	0 (0.0)	30 (100.0)
Nalidixic acid	29 (96.7)	0 (0.0)	1 (3.3)
Streptomycin	2 (6.7)	0 (0.0)	28 (93.3)
Azithromycin	26 (86.7)	0 (0.0)	4 (13.3)
Amoxicillin/clavulanic acid	0 (0.0)	0 (0.0)	30 (100.0)
Multidrug resistance	26 (86.7)		

### Phylogenetic analyses

**Fig. 1:**
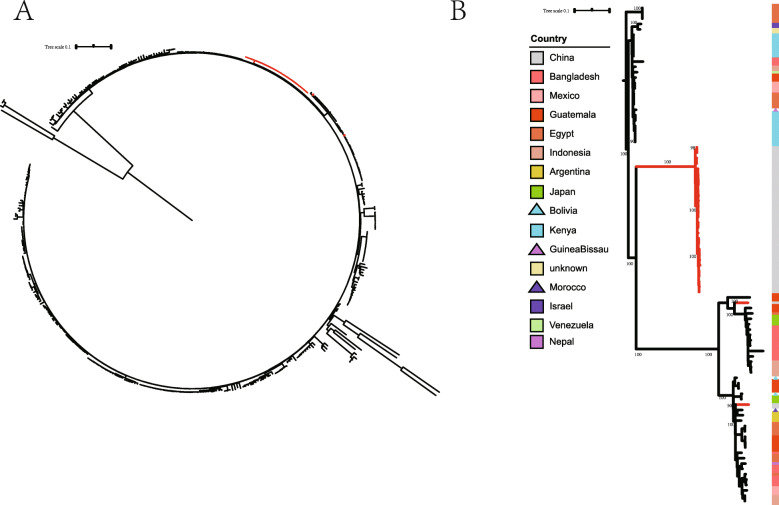
Phylogenetic tree of ETEC strains. Maximum likelihood phylogeny was estimated using RAxML (v8.2.4). The branch where the self-tested strains are located was marked in red color. **a** Complete phylogenetic tree of 392 strains. **b** The branch of the self-tested strains and its adjacent phylogenetic tree branch

We performed phylogenetic analysis of 392 strains and generated a phylogenetic tree with 143,427 SNP loci (Fig. 1a). Phylogenetic tree showed that 28 of the 30 self-tested strains clustered closely and formed an independent branch, which contained 26 strains resistant to azithromycin. This independent branch is relatively close to the strains from Africa, and there may be a transmission relationship between them. Only XHES16009 and FXEC18065 strains are located in other branches, forming another large branch with other ETEC O6 strains (Fig. 1b). The similarity among Chinese strains was 98.42–99.99%, and that between Chinese strains and foreign strains was 97.57–99.73% (Additional file 2 Table S2).

### Genotypic characterization of antimicrobial resistance

**Fig. 2:**
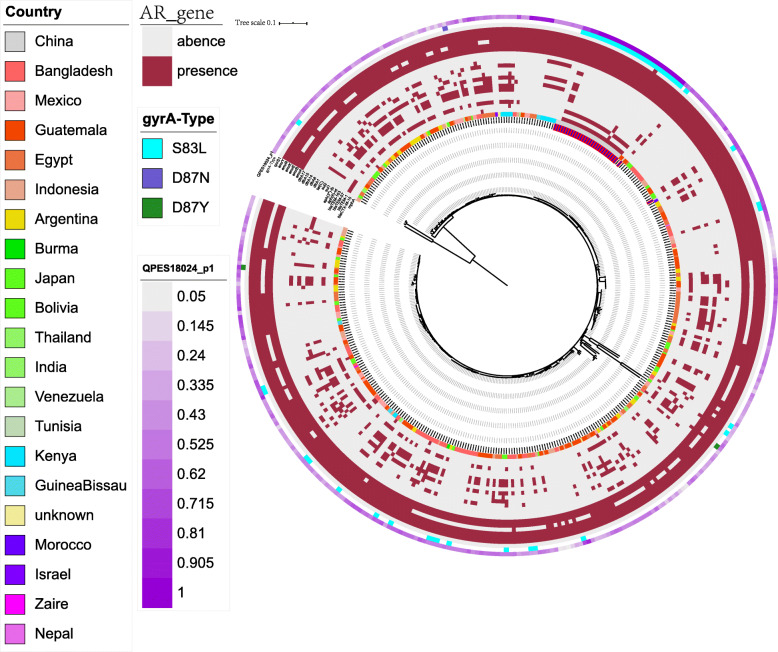
AMR gene groups detected in each genome sequence at more than 70% coverage and 80% identity using BLAST (BLASTn). Presence and absence of AMR genes were represented by dark red and light grey colors, respectively. Presence of the *gyrA* (Ser83Leu), *gyrA* (Asn87Asx) and *gyrA* (Asn87Tyr) point mutations were represented by light blue, dark blue and dark green colors, respectively. Purple with different color depths represents the strain’s coverage of plasmid pQPES18024_1

We performed antimicrobial resistance genes analysis on 392 strains, which were extracted from whole genome sequencing (WGS) analysis. The results shown in Fig. 2 indicate that the 26 self-tested azithromycin resistant strains all carried the azithromycin resistance gene *mphA* but no other azithromycin resistance genes such as *ereA*, *ereB* and *erm* family genes. While only 9/362 strains downloaded from the NCBI carry the *mphA* gene, indicating its rareness in the strains of other countries. Interestingly, a higher percentage of mutations in the *gyrA* gene (29/30) was found in the 30 sampling strains when compared with that in the 362 NCBI downloaded strains, which owns a very low percentage (24/362) of *gyrA* mutation. Besides, some beta-lactam resistance genes (*bla*CTX-M-15, *bla*TEM-1, *bla*TEM-57, *bla*TEM-163) and aminoglycoside resistance genes (*aph (6)-Id* and *aph (3″)-Ib*) were identified among these isolates as detailed in Fig. 2 and supplemental material. Additional AMR genes (*emr* family genes, *dfrA* family genes and *sul2*) were also identified among these isolates.

### S1 nuclease pulsed-field gel electrophoresis (S1-PFGE) and southern blot analysis

Analysis of S1-PFGE revealed that 30 ETEC O6 strains carried 1–4 visualize big size plasmids, most of which contained two (Additional file 3 Figure S1). The cluster analysis results of plasmid profiles showed that the 22 strains with 100% identical S1-PFGE bands constituted the largest bunch and they carried plasmids sizes of approximately 103 kb and 75 kb. In addition, the QPES18030 and XHES18006 strains carried three visualize big size plasmids, including two plasmids of 103 kb and 75 kb in size. The 24 strains carrying 103 kb and 75 kb plasmids also carried *mphA* gene, and 8 of them were selected for further Southern blot analysis. Southern hybridization with the *mphA*- specific probe showed that the *mphA* gene was located on 103 kb plasmid (Additional file 4 Figure S2).

### Complete sequence of plasmid harboring mphA

**Fig. 3:**
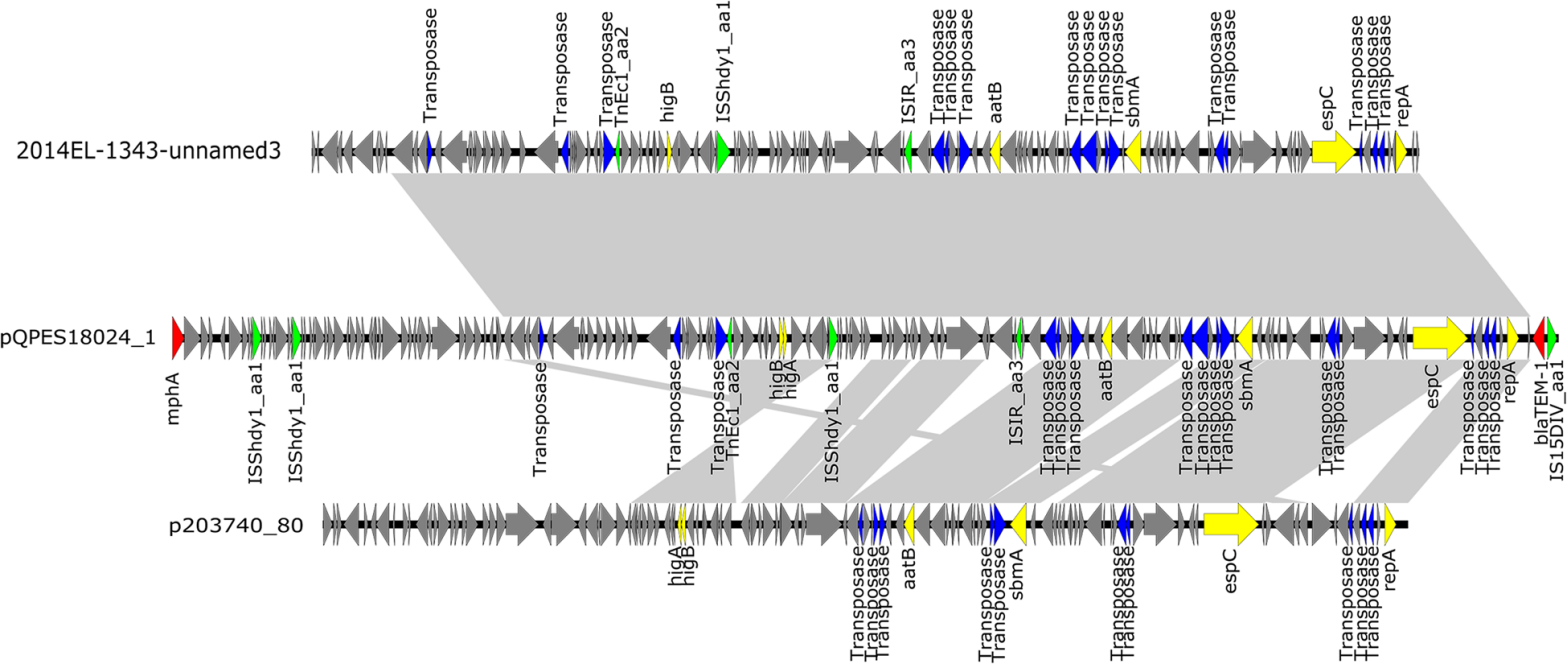
Alignment of 2014EL-1343-unnamed3, pQPES18024_1, and p203740_80. Block arrows indicate confirmed or putative open reading frames (ORFs) and their orientations. Arrow size is proportional to predicted ORF length. Resistance genes are indicated by red arrows, insert sequences are indicated by green arrows and transposases are indicated by blue arrows

The plasmid has a size of 103,206 bp and was named as pQPES18024_1, which belongs to IncFII (pCoo). It shared closest homology (at 79% coverage and a 99.84% identity) to plasmid 2014EL-1343-2_unnamed3 (GenBank accession no. CP024231.1), isolated from *E.coli*. The plasmid harbored two antibiotic resistance genes, including *mphA* and *bla*TEM-1. In addition, *mphA* is close to *bla*TEM-1 with only IS*15DIV_aa1* between them (Fig. 3). The results of plasmid coverage showed that there were 27 strains containing the plasmid, including 26 strains of the self-tested strains and 1 strain that downloaded the genomic sequence from NCBI (Fig. 2).

## Discussion

Deaths from diarrhea in children are still a health challenge worldwide, with particular concerns in low- and middle-income countries [17]. *E.coli* play an important role in the death of children by diarrhea, being involved in more than 120,000 deaths annually of children under 5 years old [17, 18]. The treatment for less severe diarrhea is often to replace lost fluids and salts with oral rehydration saline solutions to reduce the risk of dehydration, while patients with severe diarrhea are often treated with antibiotics. However, antibiotic resistance is rapidly increasing due to their commonly use, and new and alternative treatment antibacterial drugs such as azithromycin have been proposed [9, 19]. In fact, azithromycin treatment may cause *E. coli* to reduce Stx production in vivo and in vitro [20]. According to current researches and reports, *E. coli* resistance to azithromycin is generally less than 30%, and most results show that the resistance rate is between 10 and 20% [9]. However, in this study, we reported that a high proportion (approximately 86.7%) of azithromycin-resistant ETEC O6 strains was prevalent in Shanghai, China. To our knowledge, this is the first study to report a high proportion of azithromycin resistance in a particular ETEC serogroup due to a specific plasmid carrying *mphA*.

Shanghai is an important municipality of China with 6340.5 km^2^ and a population of 24.278 million. According to previous research, infectious diarrhea caused by ETEC in Shanghai mainly occurs in adults 20 to 60 years old, and July to August is the peak period of ETEC infection [21]. We investigated adults aged 20–60 who were infected with ETEC and had symptoms of diarrhea in July or August. From the regional perspective, azithromycin-resistant ETEC O6 strains appeared in six distinct districts. In terms of time, the resistance rates of azithromycin in strains isolated in different years were 50.0% (3/6) in 2016, 100.0% (8/8) in 2017 and 93.8% (15/16) in 2018. Phylogenetic analysis showed that 28 of the 30 strains self-tested were closely clustered to form a new branch, indicating that a new dominant clone had been formed locally. Of these strains, 26/28 carried *mphA* except two isolated in 2016, indicating the *mphA*-carrying part of the clone’s rapid spread in local areas.

Besides azithromycin, the vast majority of these strains are also resistant to nalidixic acid and ampicillin, and a few resistant to tetracycline, gentamicin, streptomycin, trimethoprim and sulfisoxazole. This is consistent with the results of other studies [7, 22]. It is worrying that ampicillin is still employed as one of the first line treatments in most low and middle-income countries [19, 23]. Only one of these strains is sensitive to 14 different antibiotic agents, which indicates the severity of antibiotic resistance of ETEC strains in China. The analysis of antibiotic resistance genes showed that 26 strains resistant to azithromycin all carried the *mphA* gene, and none carried the *ere* family genes and the *erm* family genes, indicating that *mphA* is an important reason of these strains resistance to azithromycin. The macrolide-resistant phosphotransferase-encoding *mphA* gene is the most common azithromycin resistance gene detected in *E. coli* [24]. Studies have shown that the presence of the *mphA* is currently the most important mechanism for high resistance to azithromycin, and 93% of the strains with MIC values above 32 mg/L [10].

The *mphA* can exist on chromosomes and on plasmids, but *E. coli* is often carried in the form of plasmids, allowing it to spread widely across this species and species closely related to its phylogenetic evolution. The *mphA* has been reported to be located on various plasmids, such as pSH15sh99, pT5282 and pEQ1 [25–27]. In this study, *mphA* was located on a 103 kb plasmid. Compared with other plasmid data in the NCBI database, other plasmids can cover only up to 79% of the plasmid. The plasmid carries two resistance genes, *mphA* and *bla*TEM-1, which confer resistance to azithromycin and ampicillin in strains carrying the plasmid. The most common plasmid carrying *mphA* is in the form of IS*26*-*mphA*-*mrx*-*mphR(A)*-IS*6100* units, but it is present in the form of IS*15DIV_aa1*-*mphA* units in this plasmid [28, 29]. Our research suggested that a plasmid carries the *mphA* gene in a novel way, and this plasmid appeared in large numbers of ETEC O6 stains in Shanghai in a short period.

## Conclusions

The present data demonstrate the presence of a high prevalence of azithromycin-resistant ETEC O6 strains in Shanghai of China. These strains formed an independent dominant clone in the phylogenetic tree. The main cause of azithromycin resistance is the emergence of a novel *mphA*-carrying plasmid, which has become widespread in the ETEC O6 strains in a short period of time. It is particularly important to continuously monitor the changes in antibiotic resistance patterns and resistance mechanisms of *E. coli* strains, especially the resistance mechanism to azithromycin.

## Methods

### Bacterial strains

The study subjects were ETEC cases of diarrhea, reported from the 2016–2018 Shanghai Diarrhea Surveillance Network (22 sentinel hospitals) and the Foodborne Surveillance Network (26 sentinel hospitals). The Surveillance Networks detected 176, 205 and 163 ETEC cases in 2016, 2017 and 2018, respectively. Sampling method: Cases were taken from selected surveillance sites, which surveillance sites required 2 and more ETEC cases each in July and August for 3 consecutive years. ETEC cases are defined as adults between the ages of 20 and 60 who have a single ETEC infection in July or August and are confirmed by a professional laboratory. Then, we performed serogroup identification, and the ETEC serogroup O6 strains were identified as the research objects.

### Serogrouping and enterotoxin type

Serogroup identification used the slide agglutination method. Pure cultures were heated directly or at 100 °C for 30 min to destroy the capsular (K) antigen. The identification of serogroup first follows the principle of multivalent and then monovalent, and the results are determined by observing the strong agglutination reaction with the naked eye. Serogroup identification results were also reviewed in serotypeFinder 2.0 (https://cge.cbs.dtu.dk/services/SerotypeFinder/) using sequencing data. Enterotoxin type were identified using the DEC Multiplex PCR Diagnostic Kit (Statens Serum Institut, Copenhagen, Denmark), and DNA extraction, amplification, electrophoresis, and result determination were performed in accordance with the instructions of the instruction manual.

### Antimicrobial susceptibility testing

Antimicrobial susceptibility testing was performed by broth microdilution in Sensititre Gram Negative AST Plates for E.coli & Salmonella (Thermo Fisher Scientific, Inc., West Sussex, UK) according to the methods of the Clinical and Laboratory Standards Institute (CLSI) [30]. The drug-sensitive test plate contained 14 different antibiotic agents: ceftriaxone (CRO), tetracycline (TET), ceftiofur (XNL), cefoxitin (FOX), gentamicin (GEN), ampicillin (AMP), chloramphenicol (CHL), ciprofloxacin (CIP), trimethoprim/sulfamethoxazole (SXT), sulfisoxazole (FIS), nalidixic acid (NAL), streptomycin (STR), azithromycin (AZI), and amoxicillin/clavulanic acid 2:1 ratio (AUG2). An *E. coli* ATCC 25922 strain was used for quality control. The results were interpreted based on the CLSI criteria (CLSI, 2019).

### Whole-genome sequencing and bioinformatics analysis

Whole-genome sequencing (WGS) was performed on all 30 ETEC O6 isolates. Genomic DNA was isolated from overnight cultures using the QIAamp DNA Mini Kit (Qiagen, Hilden, Germany) and paired-end sequences were generated using the Illumina MiSeq platform. Sequence reads were assembled into draft continuous sequences (contigs) using SPAdes software (v3.6.2) [31].

Based on previous studies on the global distribution of the ETEC clade by Astrid von Mentzer et al., we downloaded the assembled data of 362 ETEC strains involved in the article from the NCBI database [4]. *E. coli* strain Sakai was used as the reference for comparison. Reads mapping was performed using BWA (v0.7.12). SNPs were identified using SAMtools (v1.3) [32]. Chromosomal SNP alleles were concatenated for each strain to generate a multiple alignment of all SNPs and used RAxML (v8.2.4) [33] with a general time reversible (GTR) model and a gamma distribution to construct a maximum likelihood phylogenetic tree. ANIs among China strains and ANIs between China strains and foreign strains were calculated using JSpeciesWS [34] to evaluate the genome similarity.

All 392 strains in the phylogenetic analysis were analyzed for resistance genes. The presence of antimicrobial resistance (AMR) genes was predicted using the Resistance Gene Identifier (RGI) application of comprehensive antibiotic resistance database (CARD) [35], and AMR gene groups detected in each genome sequence at more than 70% coverage and 80% identity using BLAST (BLASTn). A heat map was drawn using ITOL (http://itol.embl.de) [36].

### S1-PFGE and southern blot analysis

S1-PFGE and Southern blot analysis were performed to estimate the size of the plasmid carrying the *mphA* gene. In brief, the isolates were embedded in 10 g/L Seakem Gold gel, digested with endonuclease S1 nuclease (TakaRa, Dalian, China) for 15 min at 37 °C. Chromosomal DNA of *Salmonella serotype Braenderup* (H9812) digested with *X*baI was used as reference markers. Electrophoresis was run on a CHEF MAPPER variable angle system (Bio-Rad, California, America) with the parameters set at 0.22 s ~ 26.29 s at 6 V/cm for 15 h. Images were captured using a Gel Doc 2000 system (Bio-Rad) and converted to TIF format files for further analysis. Captured images were imported into the BioNumerics software version 6.0 database for processing and analysis. Cluster analysis used an unweighted pair group method with arithmetic mean (UPGMA). DNA fragments are transferred horizontally to nylon membrane (Millipore, USA) and hybridized with digoxin-labeled probes obtained by PCR amplification.

### Nanopore sequencing and plasmid bioinformatics analysis

As the results of southern blot analysis showed that *mphA* were all located on plasmids of the same size, one strain QPES18024 was randomly selected for nanopore sequencing. Genomic DNA was extracted by Qiagen Genomic DNA extraction kit (Qiagen, Hilden, Germany) according to the standard operating procedure provided by the manufacturer. Sequences were generated using Nanopore GridION X5 sequencer (Oxford Nanopore Technologies, Oxford, UK). Sequence reads were assembled into draft continuous sequences (contigs) using Flye [37] and polished with short reads [38]. Blastn was used to compare the genome sequence to the plasmid library. When the .hit length accounts for more than 20% of the length and the sequence size is less than 1 M, the sequence is considered to be a plasmid.

The plasmid sequence containing the *mphA* gene was selected for plasmid bioinformatics analysis. The RAST [39] annotation pipeline was chosen to perform rapid annotation of the plasmids. Antibiotic resistance genes were identified using Resfinder (https://cge.cbs.dtu.dk/services/ResFinderFG/), the insertion sequences (ISs) were identified by using ISfinder (https://www-is.biotoul.fr/search.php), and the replicons genotypes were identified using PlasmidFinder (https://cge.cbs.dtu.dk/services/PlasmidFinder/). Sequence assemblies were queried against this database with BLASTn (requiring > 95% sequence identity over > 90% of the reference sequence length). The sequence of pQPES18024_1 was BLAST and share homology with the NCBI plasmid database.

## Supplementary information

**Additional file 1: Table S1.** Isolation year and regional distribution of the strains

**Additional file 2: Table S2.** ANI values between the different countries.

**Additional file 3: Figure S1.** A UPGMA dendrogram of the 30 ETEC strains plasmid profile (Optimization:0.5%, Tolerance:1.2%).

**Additional file 4: Figure S2.** Plasmid profile and Southern blot hybridization of the eight ETEC isolates. Lane 1, *E. coli* BSES17045; lane 2, *E. coli* QPES18026; lane 3, *E. coli* QPES18024; lane 4, *E. coli* QPES18030; lane 5, *E. coli* XHES18005; lane 6, *E. coli* XHES18006; lane 7, *E. coli* FXEC18040; lane 8, *E. coli* FXEC18064. Salmonella H9812 served as the DNA marker.

**Additional file 5: Figure S3.** AMR gene groups detected in each genome sequence at more than 70% coverage and 80% identity using BLAST (BLASTn). Presence and absence of AMR genes were represented by dark red and light grey colors, respectively. Presence of the gyrA (Ser83Leu), gyrA (Asn87Asx) and gyrA (Asn87Tyr) point mutations were represented by light blue, dark blue and dark green colors, respectively.

## Data Availability

All data generated or analyzed during this study are included in this published article and its supplementary information files. The complete nucleotide sequences of 30 ETEC serogroup O6 strains were submitted to GenBank of NCBI under the accession number PRJNA 634935. The datasets used and/or analyzed during the current study are also available from the corresponding author on reasonable request.

## Abbreviations

AMP: Ampicillin
AMR: Antimicrobial Resistance
AUG2: Amoxicillin/Clavulanic acid 2:1 ratio
AZI: Azithromycin
CARD: Comprehensive Antibiotic Resistance Database
CFs: Colonization Factors
CHL: Chloramphenicol
CIP: Ciprofloxacin
CRO: Ceftriaxone
ETEC: Enterotoxigenic *Escherichia coli*
FIS: Sulfisoxazole
FOX: Cefoxitin
GEN: Gentamicin
ISs: Insertion sequences
LT: Heat-labile Toxin
MDR: Multidrug-Resistant
NAL: Nalidixic acid
RGI: Resistance Gene Identifier
S1-PFGE: S1 Nuclease Pulsed-field Gel Electrophoresis
ST: Heat-stable Toxin
STR: Streptomycin
SXT: Trimethoprim/Sulfamethoxazole
TET: Tetracycline
XNL: Ceftiofur
UPGMA: Unweighted Pair Group Method with Arithmetic mean
WGS: Whole Genome Sequencing
