# Supplementary material for: A new plasmid carrying mphA causes prevalence of azithromycin resistance in enterotoxigenic Escherichia coli serogroup O6
Source: BMC Microbiol. 2020 Aug 11;20:247. doi: 10.1186/s12866-020-01927-z (PMC7418381; doi:10.1186/s12866-020-01927-z)
Supplement: Supplementary file 1 — Additional file 1: Table S1. Isolation year and regional distribution of the strains [file 12866_2020_1927_MOESM1_ESM.docx]

**Additional file 1: Table S1.** Isolation year and regional distribution of the strains

| Isolation | Region | | | | | |
| --- | --- | --- | --- | --- | --- | --- |
| year | Fengxian | Pudong | Baoshan | Xuhui | Putuo | Qingpu |
| 2016 | 2 | 1 | 0 | 1 | 0 | 2 |
| 2017 | 1 | 2 | 4 | 0 | 0 | 1 |
| 2018 | 3 | 2 | 2 | 2 | 3 | 4 |
