# Supplementary material for: A new plasmid carrying mphA causes prevalence of azithromycin resistance in enterotoxigenic Escherichia coli serogroup O6
Source: BMC Microbiol. 2020 Aug 11;20:247. doi: 10.1186/s12866-020-01927-z (PMC7418381; doi:10.1186/s12866-020-01927-z)
Supplement: Supplementary file 2 — Additional file 2: Table S2. ANI values between the different countries. [file 12866_2020_1927_MOESM2_ESM.docx]

**Additional file 1: Table S2.** ANI values between the different countries.

| country | China | Egypt | Bangladesh | Indonesia | Mexico | Argentina | Bolivia | Guatemala |
| --- | --- | --- | --- | --- | --- | --- | --- | --- |
| China | * | 97.89-97.97 | 97.89-97.92 | 98.16-98.35 | 98.39-98.55 | 97.89-97.92 | 98.79-98.82 | 98.59-98.85 |
| Egypt | 97.57-97.88 | * | 98.34-98.36 | 98.19-98.53 | 97.83-97.85 | 97.79-98.00 | 97.86-97.90 | 97.89-97.96 |
| Bangladesh | 97.68-97.71 | 98.57-98.58 | * | 98.40-98.43 | 98.00-98.22 | 97.71-98.02 | 97.92-97.94 | 97.86-97.93 |
| Indonesia | 98.07-98.59 | 98.08-98.43 | 97.68-98.44 | * | 98.12-98.38 | 97.82-98.12 | 98.28-98.57 | 98.30-98.72 |
| Mexico | 98.39-98.56 | 97.93-97.96 | 97.74-97.96 | 98.05-98.12 | * | 97.67-97.80 | 98.49-98.84 | 98.41-98.92 |
| Argentina | 98.07-98.77 | 98.14-98.46 | 97.97-98.38 | 98.19-98.36 | 97.92-98.64 | * | 98.12-99.39 | 98.09-99.37 |
| Bolivia | 98.69-98.73 | 97.99-98.06 | 97.89-98.07 | 98.22-98.25 | 98.55-98.59 | 97.80-97.87 | * | 98.87-99.73 |
| Guatemala | 98.80-98.82 | 97.98-98.03 | 97.90-98.05 | 98.23-98.27 | 98.54-98.59 | 97.81-97.83 | 99.69-99.71 | * |
