# Supplementary figures and images for: A new plasmid carrying mphA causes prevalence of azithromycin resistance in enterotoxigenic Escherichia coli serogroup O6
Source: BMC Microbiol. 2020 Aug 11;20:247. doi: 10.1186/s12866-020-01927-z (PMC7418381; doi:10.1186/s12866-020-01927-z)

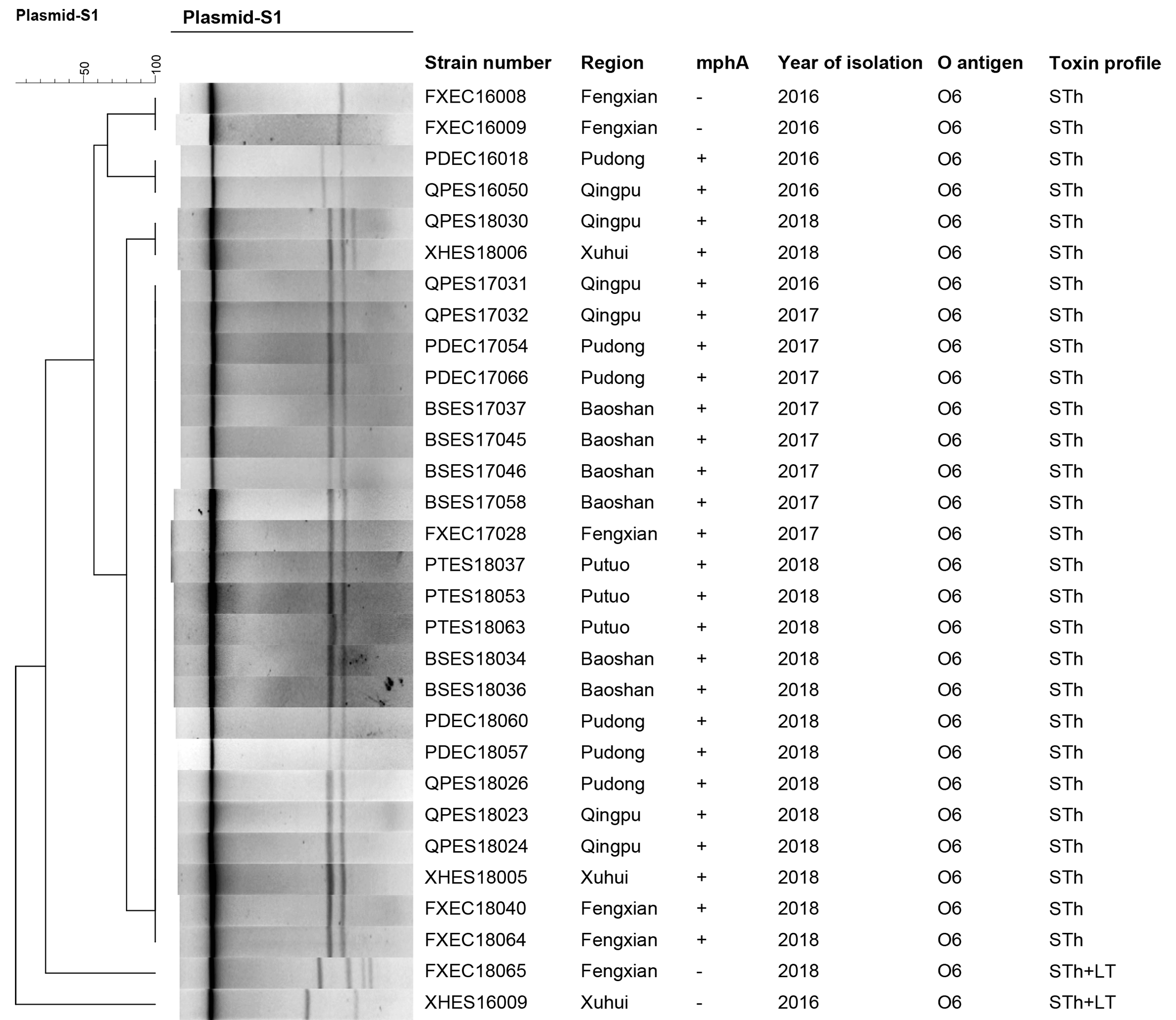

Supplement: Supplementary file 3 — Additional file 3: Figure S1. A UPGMA dendrogram of the 30 ETEC strains plasmid profile (Optimization:0.5%, Tolerance:1.2%). [file 12866_2020_1927_MOESM3_ESM.tif]

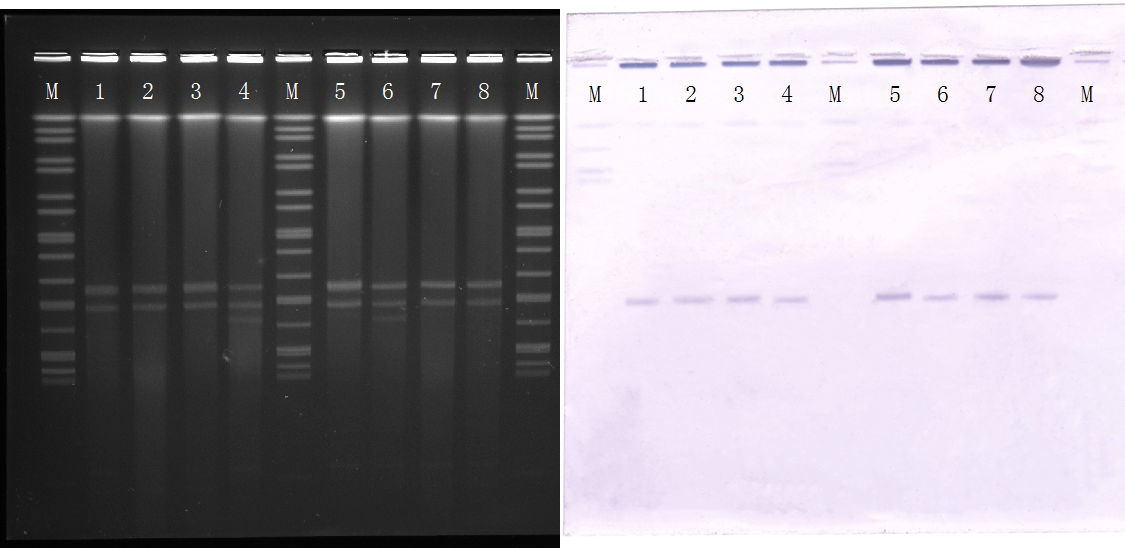

Supplement: Supplementary file 4 — Additional file 4: Figure S2. Plasmid profile and Southern blot hybridization of the eight ETEC isolates. Lane 1, E. coli BSES17045; lane 2, E. coli QPES18026; lane 3, E. coli QPES18024; lane 4, E. coli QPES18030; lane 5, E. coli XHES18005; lane 6, E. coli XHES18006; lane 7, E. coli FXEC18040; lane 8, E. coli FXEC18064. Salmonella H9812 served as the DNA marker. [file 12866_2020_1927_MOESM4_ESM.jpg]
