# Supplementary material for: A new plasmid carrying mphA causes prevalence of azithromycin resistance in enterotoxigenic Escherichia coli serogroup O6
Source: BMC Microbiol. 2020 Aug 11;20:247. doi: 10.1186/s12866-020-01927-z (PMC7418381; doi:10.1186/s12866-020-01927-z)
Supplement: Supplementary file 5 — Additional file 5: Figure S3. AMR gene groups detected in each genome sequence at more than 70% coverage and 80% identity using BLAST (BLASTn). Presence and absence of AMR genes were represented by dark red and light grey colors, respectively. Presence of the gyrA (Ser83Leu), gyrA (Asn87Asx) and gyrA (Asn87Tyr) point mutations were represented by light blue, dark blue and dark green colors, respectively. [file 12866_2020_1927_MOESM5_ESM.pdf]

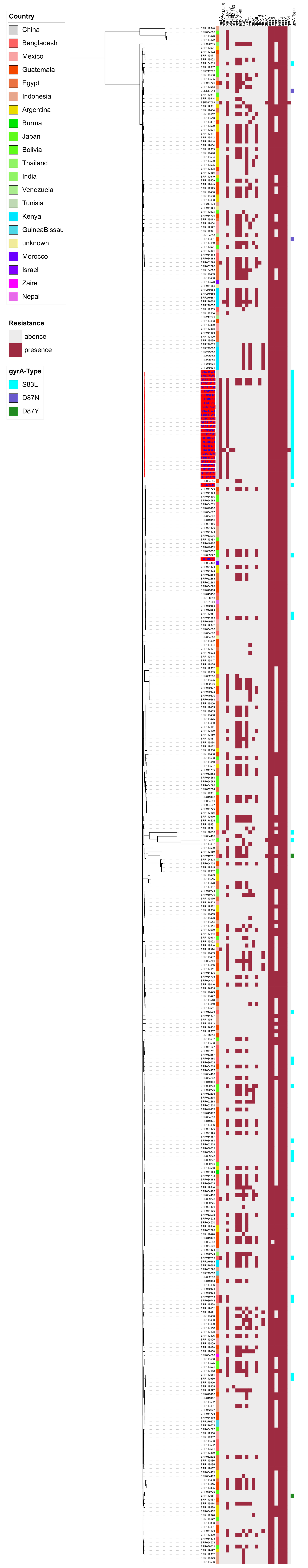

Country
China
Bangladesh
Mexico
Guatemala
Egypt
Indonesia
Argentina
Burma
Japan
Bolivia
Thailand
India
Venezuela
Tunisia
Kenya
GuineaBissau
unknown
Morocco
Israel
Zaire
Nepal
Resistance
abence
presence
gyrA-Type
S83L
D87N
D87Y
